# The role of MMP-2 and MMP-9 in the metastasis and development of hypopharyngeal carcinoma

**DOI:** 10.1016/j.bjorl.2019.10.009

**Published:** 2019-12-10

**Authors:** Zhe Song, Junfu Wang, Qinghong Su, Meng Luan, Xuemei Chen, Xiaoqun Xu

**Affiliations:** a Yantai Central Blood Station, Yantai, China; b Shandong Provincial Hospital, Shandong First Medical University, Jinan, China

**Keywords:** Hypopharyngeal carcinoma, Matrix metalloproteinase-2 (MMP-2), Matrix metalloproteinase-9 (MMP-9)

## Abstract

**Introduction:**

The role of matrix metalloproteinase-2 and 9 in the metastasis and development of hypopharyngeal carcinoma has not been clarified.

**Objectives:**

To observe the relationship between matrix metalloproteinase-2, matrix metalloproteinase-9 and the metastasis, development of hypopharyngeal carcinoma.

**Methods:**

This study included 42 hypopharyngeal cancer patients. The mRNA and protein expression levels of matrix metalloproteinase-2 and 9 in hypopharyngeal carcinoma and paracancerous tissues were detected by reverse transcription-polymerase chain reaction and Western blot.

**Results:**

Reverse transcription-polymerase chain reaction detection showed that the mRNA of matrix metalloproteinase-2 and 9 was expressed in both cancer and pericarcinoma tissues, but was almost not expressed in polypoid control tissues. The expression intensity in the cancer tissue was significantly higher than that in the pericarcinoma tissue (matrix metalloproteinase-2: t ＝ 2.529, *p* = 0.015; matrix metalloproteinase-9: t ＝ 4.781, *p* ＜ 0.001). The mRNA expression in the cancer tissue was enhanced with the increase of the tumor clinical stage (matrix metalloproteinase-2: F = 4.003, *p* = 0.026; matrix metalloproteinase-9: F = 5.501, *p* = 0.008). Its expression intensity was associated with the metastasis of lymph nodes (N staging) and increased with the degree of lymphatic metastasis (matrix metalloproteinases-2: F = 8.965, *p* = 0.005; matrix metalloproteinase-9: F = 5.420, *p* = 0.025). There was no significant change in T staging of tumor. With the increase of tumor pathological stage, the mRNA expression of matrix metalloproteinase-2 and 9 was strengthened (matrix metalloproteinase-2: F = 3.884, *p* = 0.029; matrix metalloproteinase-9: F = 3.783, *p* = 0.032). The protein expression level of matrix metalloproteinase-2 and 9 was the same as that of mRNA.

**Conclusion:**

The expression of matrix metalloproteinase-2 and 9 in hypopharyngeal carcinoma was significantly higher than that in pericarcinoma tissue, and it was enhanced with the increase of clinical stage. The expression level was related to lymph node metastasis and tumor pathological stage. Thus, matrix metalloproteinase-2 and 9 may be involved in the occurrence, development, invasion and metastasis of hypopharyngeal carcinoma through a variety of mechanisms.

## Introduction

Head and neck tumor is one of the main cancers endangering human health, ranking the tenth most common cancer in the world.^1^ Hypopharyngeal carcinoma is one of the most common types of head and neck tumors, which remains a highly lethal disease and serious threat to human life. Despite improvements in diagnosis and treatment of hypopharyngeal cancer, including surgery, irradiation and chemotherapy, the prognosis is not ideal.^2^, ^3^

To this end, the invasion and metastasis of hypopharyngeal cancer, like most human cancers, greatly contribute to cancer-related mortality. Tumor invasion and metastasis are a complex and multi-step continuous process involving various molecules, especially matrix metalloproteinases (MMPs).^4^ Matrix metalloproteinases, which represent the most prominent family of proteinases associated with tumorigenesis, are a family of zinc-dependent endopeptidases.^5^ The degradation of basement membrane and extracellular matrix (ECM) by MMPs facilitates tumor cell invasion and proliferation in the metastatic environment.^6^ The basement membrane and ECM, on one hand, provide substrates and nutrition for tumor growth and metastasis, but on the other hand, they are major blockades in the prevention of tumor cell invasion and metastasis.^7^ During the degradation of the ECM and basement membrane, MMPs are the most important enzymes and play a key role in this degradation process. The expression and activation of MMPs are also involved in various physiological and pathological events, such as inﬂammation, tissue fibrosis, angiogenesis, invasion, and tumor metastasis. Specifcally, MMPs selectively degrade different components of the ECM^8^ and thus regulate numerous biological events, including cell growth, inﬂammation, invasion, and angiogenesis by eliminating cell surface proteins such as the cytokine receptor, cell adhesion molecules, and urokinase receptors.^5^, ^9^, ^10^ Among all MMP members,MMP-2 and MMP-9 have been reported to correlate with tumor metastasis.^11^, ^12^

In the present study, the mRNA and protein expressions of MMP-2 and MMP-9 were analyzed at the tissue level by reverse transcription-polymerase chain reactions (RT-PCR) and western blot analysis to investigate the function and clinical significance of MMP-2 and MMP-9 in hypopharyngeal carcinoma and their involvement in hypopharyngeal carcinoma pathogenesis and metastasis.

## Methods

### Patients and samples

The present study includes 42 patients with a mean age of 59.45 ± 8.36 years (range 47–78 years) who were histologically diagnosed with hypopharyngeal carcinoma, and 8 of vocal cord polyp patients, with a mean age of 55.68 ± 8.27 years (range, 48–75 years), that served as age and gender-matched controls. The staging of the tumor was determined in accordance with the American Joint Committee on Cancer (AJCC), Tumor-Node Metastasis (TNM) classification. None of the patients had received any chemotherapy, radiation therapy or immunotherapy within 2 months prior to surgery. Patients with any other chronic diseases such as tuberculosis, diabetes, autoimmune diseases or other malignant tumors were excluded. Cancer tissues and pericancerous tissues were identified by stereoscopy and quick frozen sectioning. Two pieces of tissues were collected and snap-frozen for RNA extraction and protein preparation. Similarly, normal controls who had suffered fever or viral infection in the past 1 week, pregnancy, or recent accident were also excluded from the study. The protocol was approved by the Ethics Committee of Institute of Basic Medicine, Shandong Academy of Medical Sciences, and all participants signed informed consent. Representative samples of tumors and of normal control tissues were collected at surgery, snap-frozen immediately in Eppendorf tubes and stored at −80 °C to avoid RNA and protein degradation until sectioning for RT-PCR and western blot analysis.

### Main reagent

The total RNA extraction kit was purchased from Transgen Biotech Company (Beijing, China). M-MLV reverse transcriptase and Taq DNA polymerase were purchased from Promega Corporation (Madison, WI, USA). PCR primers for the detection of MMP-2, MMP-9 and β actin mRNA were designed using the OLIGO Primer Analysis Software, version 5.0 (NBA, Software and Research Services for Tomorrow's Discoveries, National Biosciences, Plymouth, MN, USA). The PCR oligomers were synthesized by a DNA/RNA synthesizer (Applied Biosystems) at BioSune biological technology corporation, Shanghai, China. Primer sequences are listed in Table 1. Rabbit anti-human MMP-2 monoclonal antibody and rabbit anti-human MMP-9 polyclonal antibody were purchased from Abcam Company (Cambridge, UK). Rabbit anti-actin polyclonal antibody was purchased from Santa Cruz Biotechnology, Inc. (Santa Cruz, CA, USA). Horseradish peroxidase-labeled goat anti-rabbit IgG were purchased from Beijing Zhongshan Golden Bridge Biotechnology Co., Ltd. (Beijing, China).

**Table 1:** MMP-2, MMP-9 and β-actin primer sequence for RT-PCR.

Aim gene	Oligonucleotide sequence	Product size (bp)
MMP-2	(F) 5’ TTGGCAGTGCAATACCTGAA 3’	425
	(R)5’ GAGTCCGTCCTTACCGTCAA 3’	
MMP-9	(F) 5’ CATCGTCATCCAGTTTGGTG 3’	669
	(R) 5’ CAGAAGCCCCACTTCTTGTC 3’	
β-actin	(F) 5’GTGGGCGCCCAGGCACCA3’	539
	(R) 5’CTCCTTAATGTCACGCACGATTT3’	

MMP, Matrix Metalloproteinases; F, Forward primer; R, Reverse primer.

### Reverse transcription polymerase chain reaction (RT-PCR)

The RT-PCR method was described briefly. RNA was extracted from tissues using the guanidine thiocyanate phenol-chloroform method. The quality of the RNA yield was assessed by electrophoresis on a 1.5% agarose gel in 0.5 moL Tris/Borate/EDTA buffer. The optical density of the RNA samples was measured and samples exhibiting an A260‒A280 ratio of 1.8–2.0 were used to obtain cDNA. RT-PCR was performed using a RNA PCR kit (Perkin-Elmer, Norwalk, CT, USA). The Relative Intensity (RI) of each band was determined according to the following equation: RI = the sum density of target gene**/**the sum density of β-actin. To exclude the possibility of contamination, reactions containing RT-PCR reagents including cytokine PCR primers without sample RNA were used as the negative control groups.

### Western blot assay

SDS-PAGE and immunoblotting were performed according to standard techniques. Briefly, the prepared tissues (100 mg) were lysed at 4 °c for 30 min in lysis buffer (Beijing Leagene Biotech. Co, Ltd, Beijing, China). The lysates were centrifuged at 15,000 rpm for 20 min at 4 °c to remove nuclei and undisrupted tissues. Protein concentration was determined using Bio-Rad protein assay solution (Bio-Rad Laboratories, Inc., Hercules, CA, USA) with bovine serum albumin as the standard.^13^ The protein samples were boiled for 10 min and loaded onto a 10% SDS-PAGE gel followed by electrophoresis for 2 h. The proteins were electrophoretically transferred onto a 0.22 μm nitrocellulose membrane. The nitrocellulose membrane was blocked with 5% skim milk at room temperature for 1 h, and immunoblotted with monoclonal rabbit anti-human MMP-2, polyclonal rabbit anti-human MMP-9 and β-actin primary antibodies. After the membrane was washed three times at 5 min intervals in PBS-T, the membrane was subsequently incubated with goat anti-rabbit IgG-HRP diluted to 1:2000 for 1 h at room temperature. After the membrane was washed three times at 5 min intervals in PBS-T, and the immunoblots were then visualized using a LAS4000 Chemiluminescence Imager (Fijifilm, Tokyo, Japan) with associated software. For presentation, immunoblots were opened in PhotoShop CS2 (Adobe Systems, Mountain View, CA, USA).

### Statistical analysis

To determine the levels of MMP-2 and MMP-9 in hypopharyngeal carcinoma, data analysis was performed using SPSS 17.0 statistical software (SPSS, Inc., Chicago, IL, USA). Data were presented as the mean ± standard deviation. The paired samples *t*-test was used to compare differences between hypopharyngeal carcinoma and pericarcinoma tissues. One-way analysis of variance was used to compare the differences between groups at different clinical stages, lymph node metastasis and tumor pathological stage. *p* **<** 0.05 was considered to indicate a statistically significant difference.

## Results

### Patient clinicopathological characteristics

**Figure 1:**
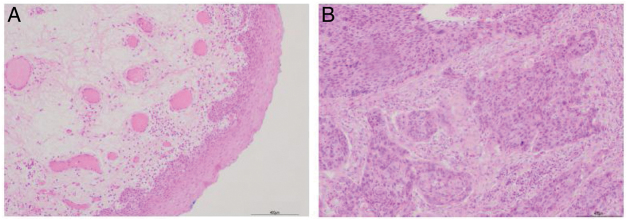
Pathological results from hypopharyngeal carcinoma patients (H&E, the scale bar is set at 400 μm). A, Pericarcinoma tissue; B, Carcinoma tissue.

Table 2 shows the descriptive characteristics of the study subjects which include 42 hypopharyngeal carcinoma patients with an average age of 59.45 ± 8.36 years (range 47–78 years), and 8 vocal cord polyp patients with an average age of 55.68 ± 8.27 years (range 48–75 years). Out of total 42 hypopharyngeal carcinoma patients, 38 (90.48%) were male and only 4 (9.52%) were female, a male to female ratio up to 9.5. In accordance with TNM classification, the majority of patients presented with large tumors (T3 + T4; 64.28%) and lymph node involvement (N+; 66.67%). The majority of patients exhibited advanced stage disease (Stage III + IV, 80.95%), while 19.05% exhibited early stage cancer (Stage I + II). All the patients were pathologically diagnosed with squamous cell carcinoma (Fig. 1). Histologically, 71.43% of patients exhibited poorly- or moderately-differentiated tumors.

**Table 2:** Clinicopathological characteristics of study subjects.

Characteristics	No (%)	
	Patients	Control
Age (yr)		
Range	47–78	48–75
Mean ± SD	59.45 ± 8.36	55.68 ± 8.27
Gender		
Male	38 (90.48)	7
Female	4 (9.52)	1
Tumor Size		
T1+T2	15 (35.72)	
T3	23 (54.76)	
T4	4 (9.52)	
Lymph node involvement		
N0	14 (33.33)	
N+	28 (66.67)	
Pathological classification		
Squamous cell carcinoma	42 (100.0)	
Histological classification		
Well differentiated	12 (28.57)	
Moderately differentiated	14 (33.33)	
Poorly differentiated	16 (38.10)	
Clinical stage		
I + II	8 (19.05)	
III	19 (45.24)	
IV	15 (35.71)

### The higher expression of MMPS in hypopharyngeal carcinoma

**Figure 2:**
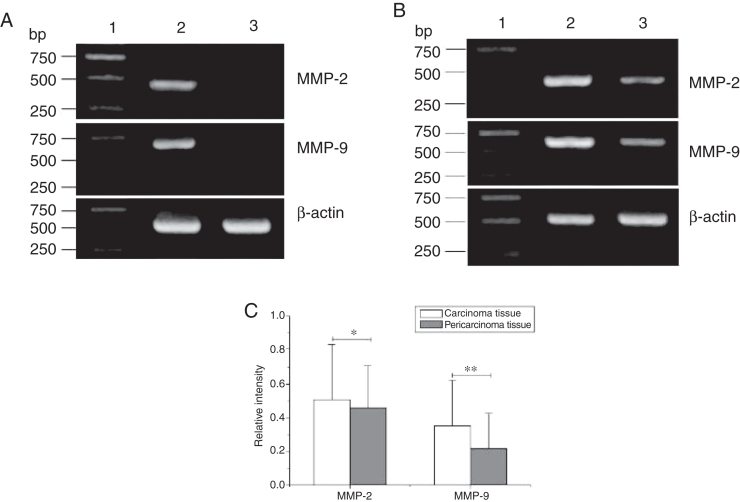
The mRNA expression of MMP-2 and MMP-9 in tumor tissues, pericarcinoma tissues and polypoid control tissues obtained from hypopharyngeal carcinoma patients and polyp of vocal cords patients. (A) Representative mRNA expression of MMP-2 and MMP-9 in tumor tissues and polypoid control tissues. Cancer tissues exhibited stronger mRNA expression of MMP-2 and MMP-9, but there was almost no expression in polypoid control tissues (lane 1, 1 kb DNA ladder; lane 2, carcinoma tissue; lane 3, polypoid control tissue). (B) Representative mRNA expression of MMP-2 and MMP-9 in tumor tissues and pericarcinoma tissues. Cancer tissues exhibited a higher level mRNA expression of MMP-2 and MMP-9 than pericarcinoma tissues (lane 1, 1 kb DNA ladder; lane 2, carcinoma tissue; lane 3, pericarcinoma tissue). (C) Relative intensity of MMP-2 and MMP-9 mRNA expression in carcinoma tissues and pericarcinoma tissues. Carcinoma tissues exhibited higher levels of MMP-2 and MMP-9 mRNA expression than pericarcinoma tissues. (*p < 0.05 and **p < 0.01).

Total RNA of 84 fresh tissues obtained from hypopharyngeal carcinoma patients and 8 vocal cord polyp tissues were prepared. The mRNA expression profiles from the aforementioned tissues were analyzed by RT-PCR. To exclude the possibility of carry-over contamination, reactions containing all RT-PCR reagents including cytokine PCR primers without sample RNA were used as negative controls. No contamination was detected. The mRNA expressions of MMP-2 and MMP-9 in tissues were analyzed. The expressing capacity (measured as Relative Intensity [RI] to the ratio of β-actin) of patients for MMP-2 and MMP-9 was higher in hypopharygeal carcinoma tissues than in pericarcinoma tissues. There was no MMP-2 and MMP-9 mRNA expression in polyp of vocal cord tissues (Fig. 2). In all 42 patients, 37 cancer tissues (88.1%) and 34 pericarcinoma tissues (81.0%) expressed MMP-2 mRNA (RI 0.509 ± 0.319 and 0.353 ± 0.268, respectively; t = 2.529, *p* = 0.015); and 35 cancer tissues (83.3%) and 30 pericarcinoma tissues (71.4%) expressed MMP-9 mRNA (RI 0.461 ± 0.249, and 0.218 ± 0.208, respectively; t = 4.781, *p* < 0.001). Cancer tissues exhibited a higher level mRNA expression of MMP-2 and MMP-9 than pericarcinoma tissues, and this difference was statistically significant.

### The expression of MMPS with tumor staging

**Figure 3:**
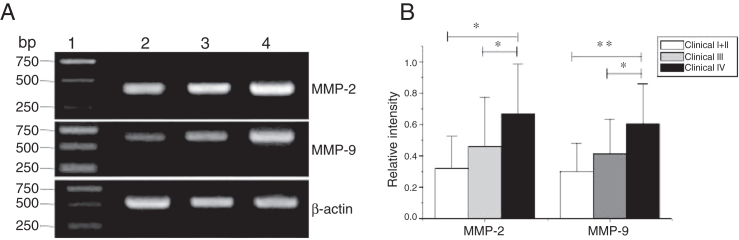
The mRNA expression of MMP-2 and MMP-9 in tumor tissues of different clinical stages obtained from hypopharyngeal carcinoma patients. (A) Representative mRNA expression of MMP-2 and MMP-9 in tumor tissues of different clinical stages. Expression of MMP-2 and MMP-9 was elevated with increasing clinical stage (lane 1, 1 kb DNA ladder; lane 2, Stage I + II patient; lane 3, Stage III patient; lane 4, stage IV patient). (B) Relative intensity of MMP-2 and MMP-9 mRNA expression in tumor tissues of different clinical stages. Advanced stage patients exhibited higher levels of MMP-2 and MMP-9 mRNA expression than early stage patients (*p < 0.05 and **p < 0.01).

**Figure 4:**
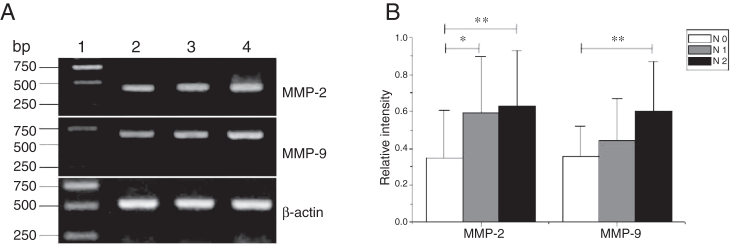
The mRNA expression of MMP-2 and MMP-9 in tumor tissues of different N stages obtained from hypopharyngeal carcinoma patients. (A) Representative mRNA expression of MMP-2 and MMP-9 in tumor tissues of different N stages. Expression of MMP-2 and MMP-9 was enhanced with increasing N stage (lane 1, 1 kb DNA ladder; lane 2, stage N0 patient; lane 3, stage N1 patient; lane 4, stage N2 patient); (B) Relative intensity of MMP-2 and MMP-9 mRNA expression in tumor tissues of different N stages. Lymphatic metastasis patients exhibited higher levels of MMP-2 and MMP-9 mRNA expression than stage N0 patients (*p < 0.05 and **p < 0.01).

**Figure 5:**
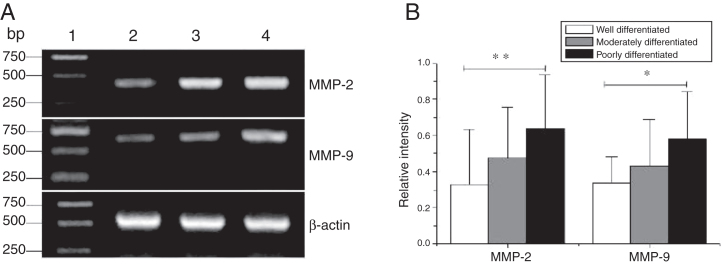
The mRNA expression of MMP-2 and MMP-9 in tumor tissues of different pathological stages obtained from hypopharyngeal carcinoma patients. (A) Representative mRNA expression of MMP-2 and MMP-9 in tumor tissues of different pathological stages. Expression of MMP-2 and MMP-9 was declined with the degree of differentiation becomeing better (lane 1, 1 kb DNA ladder; lane 2, well differentiated patient; lane 3, moderately differentiated patient; lane 4, poorly differentiated patient); (B) Relative intensity of MMP-2 and MMP-9 mRNA expression in tumor tissues of different pathological stages. Poorly differentiated patients exhibited higher levels of MMP-2 and MMP-9 mRNA expression than well differentiated patients (*p < 0.05 and **p < 0.01).

MMP-2 and MMP-9 mRNA expressions were enhanced with the increase of the tumor clinical Stages (MMP-2: F = 4.003, *p* = 0.026, MMP-9: F = 5.501, *p* = 0.008). Especially in Stage Ⅳ was obviously higher than that in Ⅰ+Ⅱ (Fig. 3). Their expression intensity were associated with the metastasis of lymph nodes (N staging) and increased with the degree of lymphatic metastasis (MMP-2: F = 4.584, *p* = 0.016; MMP-9: F = 4.643, *p* = 0.006) (Fig. 4). The expression of MMP-2 and MMP-9 in the cancer tissues is enhanced with the increase of tumor pathological stage (MMP-2: F = 3.884, *p* = 0.029, MMP-9: F = 3.783, *p* = 0.032) (Fig. 5).

### Immunoblotting revealed the protein expression levels of MMPs in hypopharyngeal carcinoma

**Figure 6:**
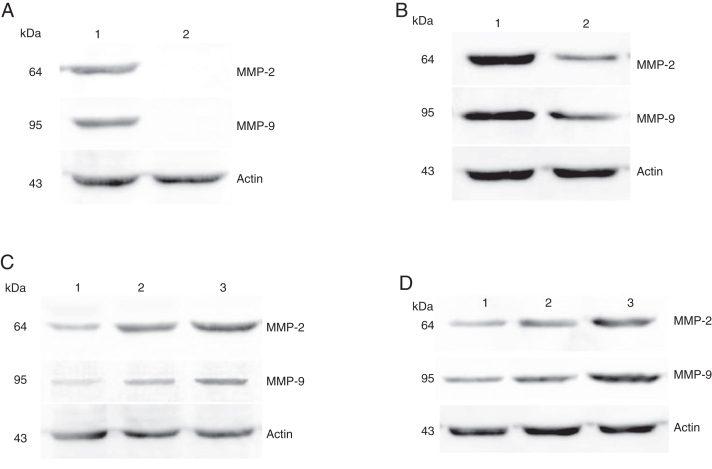
Protein expressions of MMP-2 and MMP-9 in tumor tissues and pericarcinoma tissues obtained from hypopharyngeal carcinoma patients. (A) Representative protein expression of MMP-2 and MMP-9 in tumor tissues and polypoid control tissues. Cancer tissues exhibited stronger protein expression of MMP-2 and MMP-9, but there was almost no expression in polypoid control tissues (lane 1, tumor tissue; lane 2, polypoid control tissue). (B) Representative protein expression of MMP-2 and MMP-9 in tumor tissues and pericarcinoma tissues. MMP-2 and MMP-9 protein expression were higher in tumor tissues than that in percarcinoma tissues (lane 1, tumor tissue; lane 2, pericarcinoma tissue). (C) Representative protein expression of MMP-2 nd MMP-9 in tumor tissues of different clinical stages. Expression of MMP-2 and MMP-9 protein was elevated with increasing clinical stage (lane 1, Stage I + II patient; lane 2, Stage III patient; lane 3, Stage IV patient). (D) Representative protein expression of MMP-2 and MMP-9 in tumor tissues of different N stages. Expression of MMP-2 and MMP-9 protein was enhanced with increasing N stage (lane 1, stage N0 patient; lane 2, stage N1 patient; lane 3, stage N2 patient).

The protein expressions of MMP-2 and MMP-9 were analyzed by western blot analysis. The results showed that the protein expression levels of MMP-2 and MMP-9 was consistent with the mRNA expression in hypopharyngeal carcinoma and pericarcinoma tissues. The protein expression of MMP-2 and MMP-9 in cancer tissues was higher than that in pericarcinoma tissues. There was no protein expression in polyp of vocal cord tissues (control tissues). The protein expression level of MMP-2 and MMP-9 was significantly enhanced with the increase of the tumor clinical stages and the metastasis of lymph nodes (N staging) (Fig. 6).

## Discussion

Squamous cell carcinoma of the head and neck is a significant contributor to worldwide patient morbidity and mortality. There are more than 500,000 new cases worldwide reported annually^1^, with the upper respiratory tract being the first site of contact with environmental carcinogens including certain chemicals (in cigarette smoke or alcohol), air pollutants,^14^ as well as oncogenic viruses,^15^ the incidence of head and neck cancer will probably continue to rise in the next decade.^16^ Hypopharyngeal cancer is undifferentiated Squamous Cell Carcinoma (HSCC). It represents a distinct clinical entity among other cancers of the head and neck region. It is less prevalent than other head and neck cancers, accounting for 3%–5% of all HNSCC in the region^17^. and is usually difficult to detect hypopharyngeal carcinoma at an early stage because of inconspicuous pharyngeal symptoms.^18^ In spite of considerable advances in multimodality therapy, including surgery, radiotherapy, and chemotherapy, the overall survival rate for patients with HSCC is only 15-45%.^2^, ^19^ These patients usually present late in cancer development, with 60–80% of the patients having ipsilateral nodal metastasis and up to 40% having contralateral occult nodal tumor deposits on presentation.^20^, ^21^ Either at presentation or during follow-up, patients with HSCC are usually diagnosed at a late stage, and local tumor recurrence and distant metastasis occur quite commonly after conventional therapies, which are the primary cause for poor survival of HSCC.^2^, ^19^ There is a lack of effective strategy for the best treatment,^22^ therefore treatment regimens still remain controversial. In this study, all the patients were squamous cell carcinoma, involving 66.7% patients with lymph node metastasis. 80.95% of the patients were in the advanced clinical Stage (III‒IV), moderately differentiated and poorly differentiated accounted for 71.4% of all the patients. The classification of the patients was consistent with the previously reported literature.

MMPs play a key role in ECM remodeling and are involved in a variety of processes including inflammation, migration, differentiation, angiogenesis, and fibrosis. Although the expression of some MMPs is considered to be constitutive (MMP-2) or inducible (MMP-9) in the quiescent tissue, many factors affect their synthesis. Therefore, the clinical significance of MMPs, in particular MMP-2 and MMP-9, has been revealed in many pathological conditions such as neoplasms, autoimmune diseases, and chronic inflammation.^23^, ^24^ Increased production of MMP-2 and MMP-9 appears to be a useful marker of several autoimmune disorders and neoplasms.^25^ Bo LIU et al^26^ found that Matrix Metalloproteinases (MMPs) are important in the development and expansion of tumor cells in bone metastasis and skeletal osteolysis. The degradation of extracellular matrix by MMPs facilitates tumor cell invasion and proliferation in the metastatic environment.^6^ Among all MMP members, MMP-1, 2, 3, 9 and 13 have been reported to correlate with tumor metastasis.^12^ In this study, the expression intensity of MMP-2 and MMP-9 was associated with the metastasis of lymph nodes (N staging) and increased with the degree of lymphatic metastasis. MMP-9 and MMP-2 belong to gelatinase, which is one of five groups of the MMP family, based on structure and substrate specificity.^27^ MMP-2 is secreted by tumor cells and interstitial cells in the form of a zymogen and can specifically degrade collagen IV when it is hydrolyzed and activated. Our results showed that, the mRNA and protein of MMP-2 were expressed in both cancer and pericarcinoma tissues, the expression intensity in the cancer tissue being significantly higher than that in the pericarcinoma tissue. MMP-9 enhances metastasis of tumor cells by degrading collagen proteins of the ECM after being activated by extracellular proteases under different physiological and pathological conditions. In the present study, the mRNA and protein expression of MMP-9 was strengthened with the increase of tumor pathological stage. Thus, MMP-2 and MMP-9 are important proteases that are involved in invasion and metastasis of various tumors by degrading the ECM and basement membrane.^28^ Lee et al demonstrated that inhibition of MMP-2 and MMP-9 undermines the capability of bone degradation by tumor metastasis.^29^, ^30^

## Conclusion

The expression of MMP-2 and MMP-9 in hypopharyngeal carcinoma was significantly higher than that in pericarcinoma tissue, and it was enhanced with the increase of clinical stage. The expression level was related to lymph node metastasis and tumor pathological stage. Thus, MMP-2 and MMP-9 may be involved in the occurrence, development, invasion and metastasis of hypopharyngeal carcinoma through a variety of mechanisms. Further investigations are required to determine the exact mechanisms. This may provide a new direction for the targeted therapy of hypopharyngeal carcinoma.

## Funding

This work was supported by the Natural Science Foundation of Shandong Province (grant number ZR2018HL011). Science and technology project of Shandong Academy of Medical Sciences (grant number 2016-16). Academic promotion programme of Shandong First Medical University.

## Conflicts of interest

The authors declare no conflicts of interest.
